# Molecular Detection of *Rickettsia* spp. and Other Tick-Borne Pathogens in Ticks from a Nature Reserve: Implications for Zoonotic Transmission

**DOI:** 10.3390/ani15010072

**Published:** 2024-12-31

**Authors:** Santina Di Bella, Valeria Blanda, Silvia Scibetta, Ilenia Giacchino, Antonino Gentile, Giuseppina Chiarenza, Vincenza Cannella, Giovanni Provinzano, Francesca Grippi, Annalisa Guercio

**Affiliations:** 1 Centro di Referenza Nazionale per Anaplasma, Babesia Rickettsia, Theileria (C.R.A.Ba.R.T.), Istituto Zooprofilattico Sperimentale della Sicilia “A. Mirri”, 90129 Palermo, Italy; santina.dibella@izssicilia.it (S.D.B.); antogentile1980@gmail.com (A.G.); vincenza.cannella@izssicilia.it (V.C.); annalisa.guercio@izssicilia.it (A.G.); 2 Area Diagnostica Sierologica, Istituto Zooprofilattico Sperimentale della Sicilia “A. Mirri”, 90129 Palermo, Italy; ileniajak2@gmail.com (I.G.); giuseppina.chiarenza@izssicilia.it (G.C.); francesca.grippi@izssicilia.it (F.G.); 3 Riserva Naturale Monte Pellegrino, Ente Gestore Associazione Ranger d’Italia Sezione Sicilia ODV, 90146 Palermo, Italy; riserva.montepellgrino@rangersitalia.it

**Keywords:** ticks, *Rickettsia*, tick-borne pathogens, Piroplasmids, *Coxiella burnetii*, zoonosis, nature reserve

## Abstract

Ticks are a major concern for both animal and human health, as they are primary vectors of infectious pathogens. This study focused on ticks found in a nature reserve in southern Italy, highly frequented for recreational activities and inhabited by wild boars. Using molecular techniques, 214 ticks, including questing ticks and those removed from wild boars, were examined for tick-borne pathogens (TBPs), with a focus on zoonotic pathogens. Six tick species were identified: *Hyalomma lusitanicum*, *Rhipicephalus pusillus*, *Rh. sanguineus s.l.*, *Rh. bursa*, *Rh. turanicus*, and *Dermacentor marginatus*, several of which are known vectors of zoonotic pathogens. Overall, 14% of ticks were positive for TBPs, mainly bacteria of *Rickettsia* genus. Molecular analyses detected *Rickettsia slovaca*, *R. massiliae*, *Candidatus* R. shennongii, *R. conorii*, *R. felis*, and *R. barbariae*. Additionally, single detections of *Coxiella burnetii* and *Theileria annulata* were recorded. Phylogenetic analyses were conducted on *Rickettsia* sequences. The range of ticks and TBPs present in this area highlights potential public health concerns. New associations between tick species and TBPs were documented, though vector roles need further investigation. The study highlights the importance of monitoring tick populations in both rural and urban environments to protect public health and prevent tick-borne disease spreading.

## Simple Summary

Ticks are a serious health concern for animals and humans because they spread many infectious pathogens. This study focused on ticks collected from a nature reserve in Sicily (Italy), a popular area for recreation that is also inhabited by wild boars. The analysis concerned 214 ticks, including those found in the environment and those taken from wild boars, using molecular techniques to detect tick-borne pathogens (TBPs), especially those that can infect humans. Six species of ticks were identified: *Hyalomma lusitanicum*, *Rhipicephalus pusillus*, *Rh. sanguineus s.l.*, *Rh. bursa*, *Rh. turanicus*, and *Dermacentor marginatus*. Fourteen percent of the ticks tested positive for pathogens, mostly bacteria from the *Rickettsia* genus, followed by single detections of *Coxiella burnetii* and *Theileria annulata*. Molecular identification detected *Rickettsia slovaca*, *R. massiliae*, *Candidatus* R. shennongii, *R. conorii*, *R. felis*, and *R. barbariae*. This diversity of ticks and pathogens highlights a potential risk to public health. The study found new links between specific tick species and TBPs, though further research is needed to understand these ticks’ roles as vectors. It emphasizes the need to monitor ticks in both rural and urban areas to help prevent the spread of tick-borne diseases.

## 1. Introduction

Ticks (Acari: *Ixodidae*) are among the primary vectors of infectious diseases in animals and represent a significant global threat to humans, pets, wildlife, and livestock. They play a key role in transmitting various tick-borne pathogens (TBPs), including bacteria (e.g., *Borrelia*, *Rickettsia, Anaplasma*), viruses (e.g., tick-borne encephalitis virus (TBEV)), and protozoa (e.g., *Babesia, Theileria*), some of which are also responsible for zoonotic diseases [1].

In recent years, there has been growing attention toward ticks and the pathogens they transmit, particularly those responsible for zoonotic diseases. The global increase in both TBPs and the incidence of tick-borne diseases is driven by complex, multifaceted global changes [2,3]. Several factors are contributing to the increased interaction between humans and these arthropods, including climate change [4], the rising presence of wild animals in rural and peri-urban areas [5], and the growing public interest in outdoor activities [6]. As a result, human tick-borne diseases are emerging and have become a significant public health concern [7].

Urban parks and nature reserves are heavily frequented areas for recreational and sporting activities, as well as for day trips. The Monte Pellegrino Nature Reserve is a regional protected area established in 1996, located in the northern part of Palermo (Sicily, Italy). The reserve hosts a diverse range of fauna and flora, featuring an extensive artificial forest as well as various natural habitats, contributing to significant biodiversity [8]. Additionally, the reserve serves as a popular recreational area for many inhabitants of the city. A previous study was conducted in the same area to analyze the spatial and temporal distribution of ticks in the peri-urban area. The species collected included *Ixodes ventalloi, Hyalomma lusitanicum, Rhipicephalus sanguineus, Rhipicephalus pusillus, Haemaphysalis sulcata, Dermacentor marginatus,* and *Rhipicephalus turanicus*. Many of these tick species are significant pathogen vectors, indicating the potential exposure of both animals and humans to TBPs [8]. However, a study of the pathogens present in ticks from this area has never been conducted so far.

Additionally, in recent years, a significant increase in wild boar (*Sus scrofa*) presence has been recorded. Wild boars are commonly infested with hard ticks, and their populations have been increasing across Europe since 1965 [9]. This species can serve as a reservoir for zoonotic pathogens and contribute to maintaining and spreading tick populations. Additionally, they are frequently found in close proximity to human population, as they are increasingly occupying or using urbanized areas [10].

Currently, vector surveillance and control measures remain the most effective approach to limit tick-borne diseases since effective vaccines are unavailable for most TBPs [11,12]. Monitoring tick distribution and identifying pathogens harbored by these vectors are among the most valuable strategies for reducing the risk of tick-borne diseases’ transmission.

This study aimed to investigate the presence of TBPs in questing ticks collected from a nature reserve in southern Italy frequented for recreational activities. Moreover, given the increasing reports of wild boars in this reserve, our study also investigated the presence of TBPs in ticks feeding on wild boars found as carcasses in the same reserve, located in close proximity to a densely populated area.

## 2. Materials and Methods

### 2.1. Study Area

**Figure 1:**
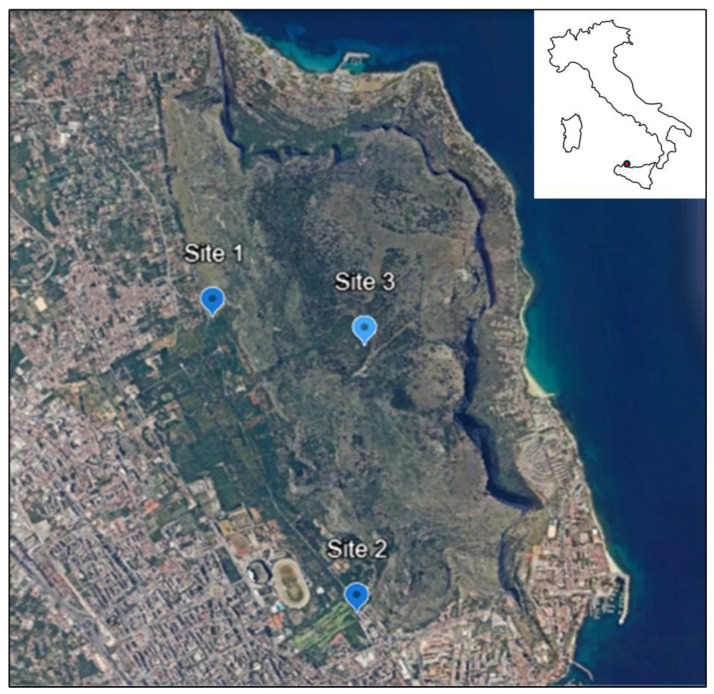
Sampling site: Site no.1 Sede Landolina (Lon 13.33809; Lat 38.17215; 76 m above sea level a.s.l.); Site no 2. Boschetto Airoldi (Lon 13.35141; Lat 38.14946; 35 m a.s.l.); Site no 3. Gorgo S. Rosalia (Lon 13.35179; Lat 38.17005; 392 m a.s.l.). The main image (Data SIO, NOAA, U.S. Navy, NGA, GEBCO) is from Google Earth.

The monitoring activities were conducted during the year 2022 within the Monte Pellegrino Nature Reserve, situated within the municipality of Palermo, approximately a few kilometers from the urban center (Figure 1). Ticks were collected from three different sites: Site no.1 Sede Landolina (Lon 13.33809; Lat 38.17215; 76 m above sea level a.s.l.); Site no 2. Boschetto Airoldi (Lon 13.35141; Lat 38.14946; 35 m a.s.l.); Site no 3. Gorgo S. Rosalia (Lon 13.35179; Lat 38.17005; 392 m a.s.l.). The collection sites are described in detail elsewhere [8].

### 2.2. Tick Collection and Morphological Identification

Sampling was conducted over a few months, in late spring–summer 2022, specifically from May to July, which coincides with the peak period for recreational activities in the nature reserve. This period aligns with the high levels of human and animal activity in the area, ensuring that the collected samples reflect the season of highest tick abundance and pathogen transmission risk. These months also correspond to the time when wild boar carcasses have been found in the area, which was a key factor in sampling design. The concurrent presence of high tick populations, reserve visitors, and wild boar carcasses during this period provides a snapshot of tick-borne pathogen circulation in a critical temporal window. 

Questing ticks were collected by dragging a blanket (1 × 1 m) over the vegetation. In addition, feeding ticks were collected from five wild boar carcasses found in the reserve. Collected specimens were placed in sterile tubes for morphological identification.

Concerning ticks from wild boars, the whole body of each animal was examined for the presence of ticks. Sterile, fine-tipped forceps were used to detach ticks from the host and the removed samples were stored in sterile tubes. On arrival at the laboratory, ticks were kept alive for a week at room temperature, so that any ingested blood was allowed to be digested, and then stored with 70% ethanol at room temperature until identification and DNA extraction.

Ticks were initially identified according to their morphological characteristics, following previously established standard taxonomic keys [13,14], with further classification according to the molecular methods described below.

### 2.3. DNA Extraction from Ticks

For molecular analysis, ticks were cut longitudinally into two halves; one half was sliced into small pieces, while the other half was stored at −20 °C as a backup. Genomic DNA extraction was then performed on each tick individually using the DNeasy Blood and Tissue Kit (Qiagen, Germany). The ticks were first mechanically disrupted in 200 µL of tissue lysis buffer provided in the kit and treated with proteinase K (100 µg/mL) at 56 °C overnight. Subsequent steps were carried out according to the manufacturer’s instructions. Extracted DNA was quantified using a NanoDrop™ 2000 spectrophotometer and then stored at −20 °C until further use.

### 2.4. Tick Molecular Identification

A fragment of Cytochrome c Oxidase subunit I (COI) gene, approximately 710 bp, was analyzed to confirm tick identification. This gene was amplified via polymerase chain reaction (PCR) using primers specific to invertebrates [15].

### 2.5. Molecular Detection of Pathogens and Sequencing

All ticks were tested for the relevant target genes, which included *ompA* and *ompB*, insertion sequence (*IS1111*), and *htpB*, *ospA*, *16S rRNA,* and *18S rRNA* genes for *Rickettsia*, *Coxiella*, *Borrelia*, *Anaplasma,* and Piroplasmids, respectively. The reactions for *Rickettsia* and *Anaplasma* were run under nested PCR settings; the other reactions were run under the real-time PCR settings. Amplification of *Coxiella burnetii* and Piroplasmids’ DNA was carried out by conventional PCRs. To better identify the species, *gltA* was used as an additional target for *Rickettsia* by performing conventional PCR on a limited number of samples. Protocols are published elsewhere [16,17,18,19,20,21,22,23,24]. All primers and probes used in this study are listed in Table 1.

Real-time PCR assays were carried out in a CFX96 Real-Time System (Bio-Rad, Hercules, CA) or QuantStudio™ 6 Pro Real-Time PCR System (Life Technologies, Thermo Fisher Scientific, Carlsbad, CA, USA). Reaction mix included 1X SsoAdvanced Universal Probes Supermix (Bio Rad Laboratories, Hercules, CA, USA), 500 nM of each primer, and 500 nM of probe, in a 20 µL total volume. The following thermal cycle conditions were used: 95 °C for 3 min, 40 cycles of 95 °C for 10 s, and 60 °C for 30 s. *Coxiella burnetii* DNA (provided by Istituto Zooprofilattico Sperimentale delle Venezie, Legnaro, Italy), *B. burgdorferi* s.l. DNA extracted from the IFA slides (Fuller Laboratories, Fullerton, CA, USA), and *B. microti* DNA provided by the WOAH Reference Center for Babesiosis were used as positive controls. Nuclease-free water was used as a negative control.

Conventional PCR assays were carried out in a SimpliAmp thermal cycler (Thermo Fisher Scientific Inc., Waltham, MA) in a final volume of 50 µL, using GoTaq G2 DNA Polymerase (Promega Italia s.r.l., Milan, Italy) with 5 µL of each DNA extract. Positive and negative controls were included in each amplification assay to evaluate the presence of appropriately sized amplicons and to rule out potential contamination. In particular, *R. conorii* DNA (Amplirun, Vircell, Granada, Spain), *A. phagocytophilum* DNA extracted from IFA slides (Fuller Laboratories, Fullerton, CA, USA), and *Babesia microti* DNA provided by the WOAH Reference Center for Babesiosis were used as positive controls. Nuclease-free water was used as a negative control. The amplicons were visualized by electrophoresis on a 2% agarose gel and observed using a SybrSafe nucleic acid staining solution under UV light. A 100 bp DNA ladder was used as a molecular-weight size marker (Amplisize^®^ Molecular Ruler, BioRad Laboratories, Hercules, CA, USA). The PCR products were quantified and sent for sequencing to Macrogen Inc. (Macrogen Europe, Amsterdam, The Netherlands). The obtained raw data, for the *ompA* and *ompB* genes, were visualized and manually edited through Chromas (Technelysium Pty Ltd., Tewantin, Australia). The fasta sequences were generated and submitted to BLAST (Basic Local Alignment Search Tool, version 2.13.0) at NCBI (National Center for Biotechnology Information) to identify the closest matching species based on sequence homology. 

### 2.6. Phylogenetic Analysis

For phylogenetic tree construction, sequence alignments were carried by MUSCLE implemented in MEGA XI [25] comparing *Rickettsia* spp. sequences indicated from BLAST analysis and closely related reference sequences selected from previous studies retrieved from NCBI molecular database [26,27]. Individual phylogenetic trees based on the *ompA* and *ompB* fragments were constructed using the maximum likelihood (ML) method and the Kimura 2-parameter model. The number of base substitutions per site between sequences, visualized by matrix, were conducted using the Kimura 2-parameter model by MEGA XI.

## 3. Results

### 3.1. Sampled Ticks

A total of 214 ticks were collected within the Monte Pellegrino Nature Reserve and analyzed for the presence of TBP DNA. In particular, 137 (64%) were free-living ticks and 77 (36%) were collected from wild boar carcasses.

Among questing ticks, 69 (50.4%) *H. lusitanicum*, 27 (19.7%) *Rh. pusillus,* 17 (12.4%) *Rh. sanguineus* s.l., 12 (8.7%) *Rh. bursa*, 9 (6.5%) *Rh. turanicus,* and 3 (2.2%) *D. marginatus* were collected from the three sampling sites. All the collected ticks were adults, comprising 48 males and 89 females.

Regarding the 77 feeding ticks collected from wild boars, all were adults, comprising 51 males and 26 females; 75 (97.4%) were *H. lusitanicum* and 2 (2.6%) were *D. marginatus*.

Details on collected ticks are reported in Table 2 and Table 3.

### 3.2. Pathogen Detection

Overall, 30 (14.0%) of the examined ticks tested positive for TBPs. Details on detected pathogens in relation to tick species and collection sites are reported in Table 2 and Table 3.

In particular, 28 (13.1%) of the examined ticks were positive for *Rickettsia* spp.; out of them, 26 were free-living and 2 were collected from wild boars.

#### 3.2.1. Pathogen Detection from Questing Ticks

Molecular identification detected *R. slovaca* in 11 ticks, of which 10 were *H. lusitanicum* and 1 was *D. marginatus*. *Rickettsia massiliae* was identified in 4 ticks: 2 *H. lusitanicum*, 1 *Rh. turanicus* and 1 *Rh. pusillus*. A total of three ticks (one *Rh. pusillus*, one *Rh. turanicus,* and one *H. lusitanicum*) resulted as positive for *Candidatus* R. shennongii. In three cases (two *H. lusitanicum* and one *Rh. turanicus*), a non-discriminative species identification between *R*. *massiliae* and *Canidatus* R. shennongii was obtained. *Rickettsia conorii* was detected in one *Rh. sanguineus* and *Rickettsia felis* in one *H. lusitanicum*. Moreover, *R. barbariae* was found in three *Rh. bursa*.

*Coxiella burnetii* was detected in one *H. lusitanicum* (0.5%).

A *H. lusitanicum* (0.5%) resulted as positive for piroplasm presence. Sequence analyses revealed the presence of *T. annulata*.

No coinfections were detected.

All the other investigated pathogens were absent.

#### 3.2.2. Pathogen Detection from Wild Boar Carcasses

Two ticks positive for *R. massiliae*, one for *H. lusitanicum,* and one for *D. marginatus* were collected from wild boars.

### 3.3. Rickettsia spp. Phylogenetic Analysis

**Figure 2:**
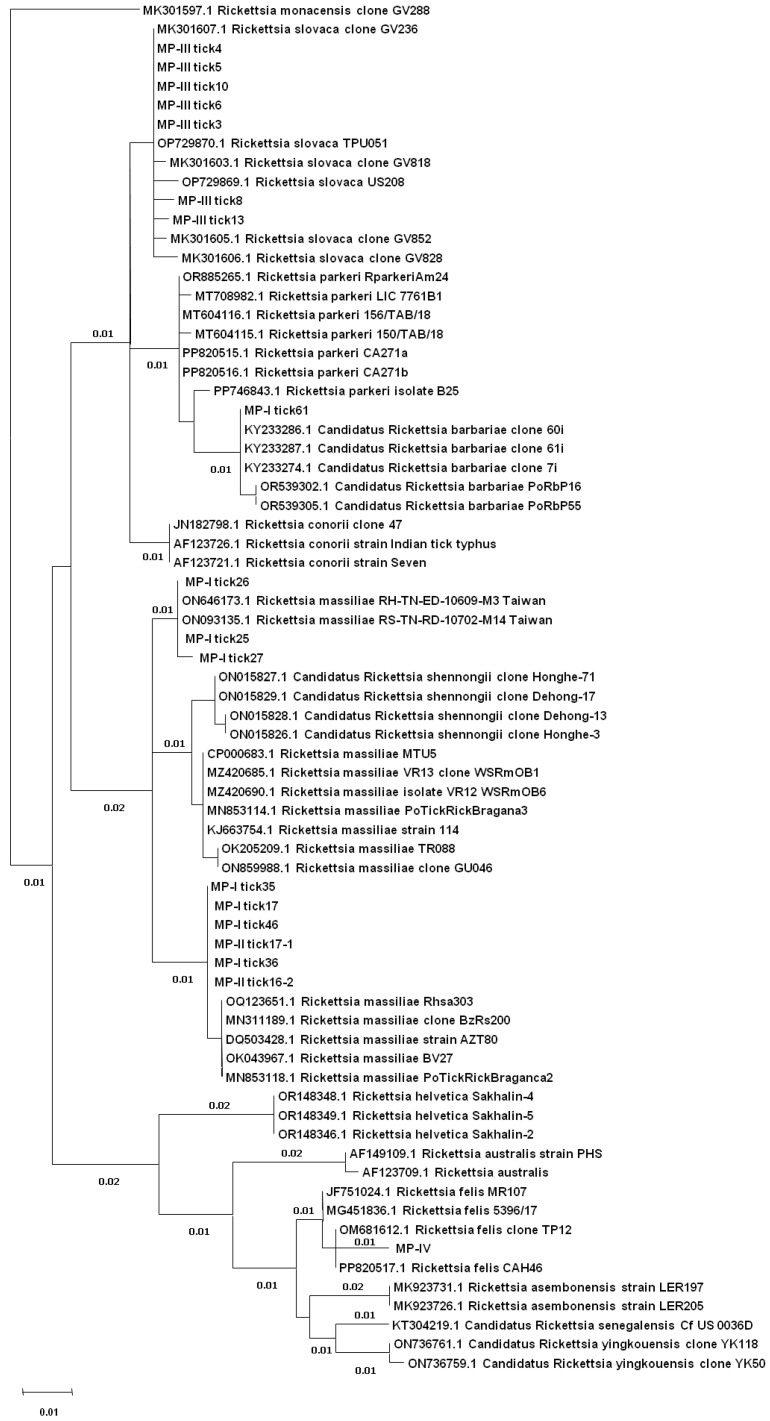
Individual phylogenetic trees based on the *ompB* fragments constructed using the ML method.

**Figure 3:**
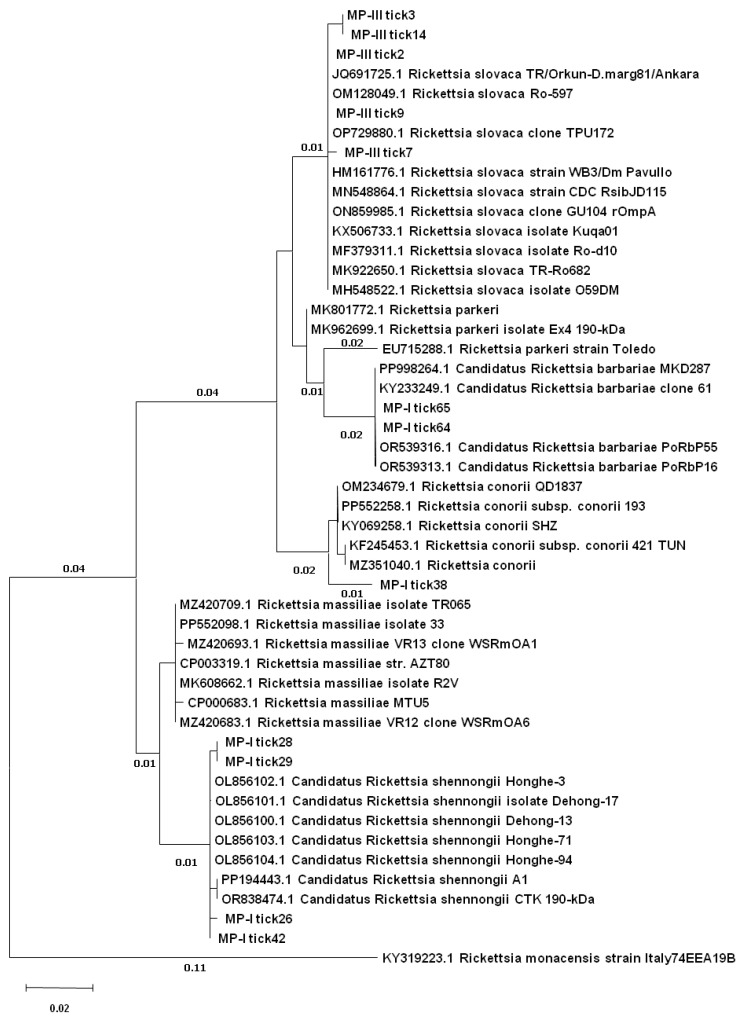
Individual phylogenetic trees based on the *ompA* fragments constructed using the ML method.

After the editing of the raw sequences, 18 sequences of good quality of the target *ompB* and 12 of the target *ompA* were retrieved. Only two samples were analyzed by both targets (MP-I tick26 and MP-III tick3). Comparing sequences to the NCBI nucleotide database by BLAST, all of them were related to *Rickettsia* spp. (Table 4). The ML trees, respectively, for *ompB* (Figure 2) and *ompA* (Figure 3), clearly showed the taxonomic relationship between the analyzed sequences.

**Figure 4:**
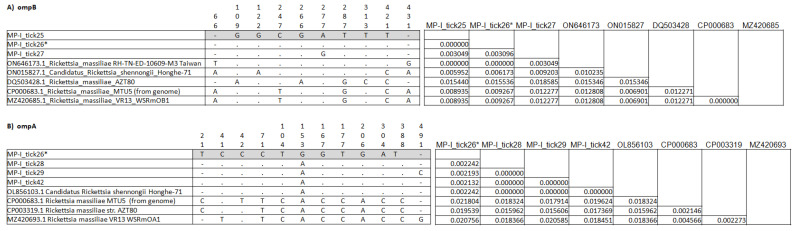
Alignments, with nucleotides’ positions of the variation points, and the matrices of evolutionary divergence between sequences related to *R. massiliae* and *Candidatus* R. shennongii: (**A**) results for *ompB*; (**B**) results for *ompA*. * Sample analyzed by both targets.

The *ompB* revealed the presence of *R. slovaca* as the most frequent in the samples (7 of 18) showing very high homology, from 99% to 100%, to an *R. slovaca* sequence obtained from a questing *Ixodes ricinus* in Spain (MK301607). *Rickettsia felis* was identified in a single sample with 99% homology to a reference sequence obtained from *Haemaphysalis intermedia* in India (OM681612). *Rickettsia barbariae,* detected in a single sample, showed total homology to a reference sequence of *R. barbariae* from *Rh. turanicus* obtained in Lebanon (KY233287). *Rickettsia massiliae* was identified in six samples, with 99% homology to a Portuguese sequence (Table 4; Figure 2), obtained from *Rh. sanguineus* (MN853118). In the other three samples, however, *ompB* did not show suitable variability to discriminate between many references of *R. massiliae* and the newly described species *Candidatus* R. shennongii (ON015827) from *Rhipicephalus haemaphysaloides* from China, which remained in doubt (Table 4; Figure 2). These sequences showed total identity only to a small group of sequences of *R. massiliae* from *Rh. haemaphysaloides* from Taiwan (ON646173), which appeared instead quite different from the other *R. massiliae* references. This appeared also evident when comparing the *ompB* nucleotide alignment and the matrix of diversity of these identities to the closest references (Figure 4A).

For one of these doubt samples, MP-I tick26, the *ompA* gene sequence was also obtained, which instead revealed a major identity (99%) towards *Candidatus* R. shennongii from *Rh. haemaphysaloides* obtained in China (OL856103). The *ompA* analysis also indicated the presence of *Candidatus* R. shennongii in the other three samples, with very high homology (99–100%) to the same sequence as for MP-I tick26 (Table 4; Figure 3). Comparing the *ompA* nucleotide alignment and the matrix of diversity of these identities to the closest references, these four samples showed the major similarity to *Candidatus* R. shennongii (Figure 4B).

The *ompA* sequences also indicated the presence of *R. slovaca* as the most frequent in the samples (5 of 12) showing very high homology, from 99% to 100%, to Spanish sequences obtained from *D. marginatus* (OP729880). In two samples, *R. barbariae* was identified with *ompA*, with total homology to a sequence (KY233249) from a *Rh. turanicus* from Lebanon (Table 4; Figure 3). One sequence of *ompA* indicated a group of *R. conorii* from China detected in *Rh. turanicus* as the closest species (KY069258), but the identity was only 98% (seven nucleotides different) (Table 4; Figure 3).

**Figure 5:**
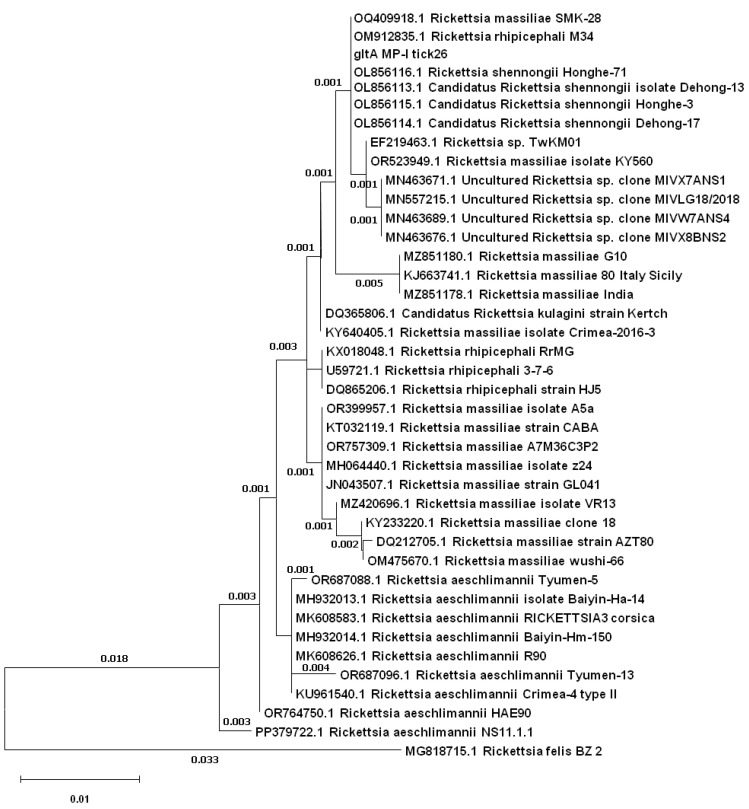
Individual phylogenetic tree based on the *gltA* fragments, constructed using the ML method, with the closest references for the sample MP-I tick26.

The target *gltA* was secondarily implemented in the analysis, just to help in clarifying the position of the doubt samples *Candidatus* R. shennongii/*R. massiliae*. To test the potential of the target in discriminating between these two species, the sample MP-I tick26 was amplified and sequenced. Results from BLAST indicated our *gltA* sequence matched 100% to *Candidatus* R. shennongii from *Rh. haemaphysaloides* (OL856116) [26] but also to an isolate of *R. rhipicephali* from *Rh. sanguineus* s. l. Iran (OM912835, Esmaili et al., unpublished) and an isolate of *R. massiliae* (OQ409918, Yarmukhamedova et al., unpublished) from Uzbekistan. Results from the phylogenetic tree, although, apparently suggested a major similarity towards *Candidatus* R. shennongii, but nucleotide dissimilarity, varying from one to three nucleotides only, indicated a very low level of divergence towards the other clusters of *R. massiliae* (Figure 5).

## 4. Discussion

This study investigated the presence of zoonotic agents in ticks collected from a nature reserve in close proximity to the urban area in southern Italy. This area was previously the focus of a more extensive study that examined the temporal and spatial distribution of tick populations [8]. In comparison to the earlier research, the present study involved a smaller number of ticks, as it primarily focused on pathogen detection rather than assessing the ecological and seasonal distribution of ticks in the area. The relative proportions of the different tick species between the two studies varied, likely due to several factors: the total number of ticks analyzed, the number of sampling sites (three in this study compared to six in the previous one), the sampling seasonality (spring–summer in this study versus year-round sampling in the previous one), and the inclusion of ticks collected not only from the environment but also from wild boars inhabiting the reserve, an aspect not addressed in the earlier study.

The study demonstrates the presence of both established and potential vector species of pathogens that affect animals and humans [8]. The following tick species were detected: *H. lusitanicum*, *Rh. sanguineus* s.l., *Rh. turanicus, Rh. bursa*, *D. marginatus, and H. marginatum*.

Among the sampled ticks, *H. lusitanicum* was the most frequently identified species. This tick predominantly parasitizes large mammals, such as domestic ruminants and pigs, and is primarily associated with the transmission of *Anaplasma* spp. and *T. annulata* [35]. Moreover, the transovarial and transstadial transmission of *C. burnetii*, the causative agent of Q fever, was documented in *H. lusitanicum*, although its vector competence for this bacterium remains to be fully established [36,37]. *Hyalomma lusitanicum* may also contribute to the transmission of spotted fever group (SFG) *Rickettsiae* [38]. Additionally, both *H. lusitanicum* and *H. marginatum* are competent vectors for the Crimean-Congo hemorrhagic fever virus (CCHFV). Autochthonous cases of CCHF have recently emerged in Europe where the virus was previously absent, with human cases reported in Greece, Bulgaria, and Spain [39].

*Rhipicephalus sanguineus* s.l., the brown dog tick, is a recognized vector for several pathogens, including *A. marginale*, *B. bigemina*, and *B. bovis* [8]. This tick parasitizes a variety of hosts, including humans, and plays a significant role in transmitting zoonotic agents such as *Rickettsia* spp. [40].

*Dermacentor marginatus,* also known as the ornate sheep tick, is widely distributed across southern Europe [41]. Although *D. marginatus* is less frequently reported to bite humans, cases of this tick being collected from human patients have been reported [42]. It is a vector of *B. caballi, T. equi,* and potentially *B. microti,* the causative agent of human babesiosis. Additionally, it transmits *R. sibirica* and *R. conorii* and is the primary vector of *R. slovaca* [9]. Furthermore, it is capable of transmitting TBEV and CCHFV.

*Rhipicephalus turanicus* is associated with the transmission of *Babesia* spp. and multiple *Rickettsia* species, including *R. monacensis, R. massiliae, R. conorii,* and *R. aeschlimannii* [6,8]. As a multi-host tick, it parasitizes sheep, goats, cattle, and horses, as well as other mammals, birds, lizards, and snakes [14].

*Rhipicephalus bursa* has been involved in the circulation of several agents including *A. marginale*, *T. equi* [43], *A. ovis*, *A. phagocytophilum* [44], *B. ovis* [45], *C. burnetii* [46], *Ehrlichia canis* [47], *R. barbariae*, *B. caballi*, *B. bigemina,* and *Theileria haneyi* [48].

Ticks collected from wild boars in this study were predominantly *H. lusitanicum*, with a smaller proportion identified as *D. marginatus*. Both species are commonly associated with wild boars [35,49,50,51]. Our findings suggest that wild boars may contribute to the maintenance of these tick species, particularly *H. lusitanicum*, in urban and peri-urban environments, potentially increasing the risk of zoonotic disease transmission [9,50].

Concerning TBPs detected in this study, a notable infection rate of *Rickettsia* spp. in ticks emerged, with 13.1% positive samples (28 out of 214). This prevalence is comparable to the findings of Scarpulla et al. [52], who reported a 12.4% infection rate in 113 ticks collected from hosts and the environment in the Latium and Tuscany regions. However, it is lower than the 20.78% infection rate documented in a similar study conducted by Ebani et al. [53] on 77 pools of three ticks each in Tuscany. To date, there is a lack of updated data on the occurrence of *Rickettsia* species in free-living ticks from Sicily.

Phylogenetic analyses identified a diverse array of *Rickettsia* species.

*Rickettsia slovaca* emerged as the most frequently detected species, confirmed through both the *ompA* and *ompB* genetic targets. Additionally, the presence of *R. felis* and *R. barbariae* highlights the diversity of *Rickettsia* species in this region, some of which may have zoonotic potential. The detection of *R. massiliae* in multiple samples further suggests that this species is prevalent in the area.

However, one of the main challenges encountered in this study was the difficulty in distinguishing between *R. massiliae* and *Candidatus* R. shennongii based on the *ompB* sequence. However, the target *ompA*, instead, seems to clearly identify these sequences as *Candidatus* R. shennongii; the inconsistency observed between the two target genes adopted in this study was considered restrictive for a univocal identification of these samples. Also, the adoption of a third target, *gltA*, to test its potential did not solve the issue. Therefore, in these samples, for two tick species *H. lusitanicum* and *Rh. turanicus*, the identification remained at the genus level for *Rickettsia* spp.

*Candidatus* R. shennongii is a species recently identified in *Rh. haemaphysaloides* ticks in China [26]. Previously, this *Rickettsia* species was named *Rickettsia* sp. and was reported in *Haemaphysalis spinigera*, *Haemaphysalis turturis*, *Haemaphysalis bandicota*, and *Rh. haemaphysaloides* from India and Taiwan [27,54]. In pet ectoparasites from India, it was called *R. massiliae* [27], highlighting the confusion in discrimination. This agent shows a broad host and geographic range and, to the best of our knowledge, it is the first time that similarity with this species has been reported in Italy. The pathogenicity of *Candidatus* R. shennongii to humans is unknown. The eventual finding of *Candidatus* R. shennongii in Italy would be the first; therefore, more targets need to be investigated to strongly support this option.

The discovery of a potential new variant of *R. conorii* also warrants additional investigation, as it could represent an unrecognized strain circulating in the region.

In the present study, *R. slovaca* was detected in both *D. marginatus* and *H. lusitanicum. Rickettsia slovaca* is the primary etiological agent responsible for TIBOLA/DEBONEL (Tick-Borne Lymphadenopathy/Dermacentor-Borne Necrosis Erythema and Lymphadenopathy) [55]. In Europe, it is primarily associated with *Dermacentor* ticks, with *D. marginatus* and *D. reticulatus* confirmed as its main vectors [56]. The strong link between *R. slovaca* and *D. marginatus* is well documented, including within Sicilian tick populations [40,57]. Furthermore, previous research highlighted the elevated risk of *R. slovaca-*related rickettsiosis in Sicily and across other regions in the Mediterranean basin [58]. *Hyalomma lusitanicum* has been considered a less efficient vector for *Rickettsia* spp. [59], although its role in harboring *R. slovaca* was observed in earlier studies [37]. However, the capacity of *H. lusitanicum* to act as a competent vector for *R. slovaca* remains unconfirmed.

The presence of *R. massiliae* was detected in *H. lusitanicum* and *Rh. sanguineus* s.l. *Rickettsia massiliae* is mainly transmitted by ticks of the *Rhipicephalus* genus. In Europe, its vectors include *Rh. sanguineus s.l.*, *Rh. bursa*, *Rh. pusillus*, and *I. ricinus* [56]. The detection of *R. massiliae* in *Rhipicephalus* ticks collected from humans was reported in Sicily [40]. The first human case of *R. massiliae* infection was reported in Sicily in a patient with Mediterranean spotted fever (MSF), and other two cases of TIBOLA/DEBONEL were attributed to *R. massiliae* in Italy [60].

In addition, two of the ticks collected from wild boars tested positive for *R. massiliae*, confirming previous reports of *R. massiliae* in *H. lusitanicum* from wild boars [35]. Unfortunately, it was not possible to test wild boar tissues for these pathogens. However, we can confidently state that wild boars carry ticks infected with zoonotic *Rickettsia* species. Considering the significant population expansion of wild boars in Sicily, as well as in many areas of Europe, coupled with their habit of venturing into urban areas in search of food, they may serve as effective sentinels for the risk of transmission of TBPs to humans in the area [9].

Another notable finding is the detection of *R. felis*, the etiological agent of flea-borne spotted fever, an emerging rickettsiosis of medical significance and a common cause of febrile illness in humans [61]. Although the cat flea (*Ctenocephalides felis*) is currently recognized as the only confirmed biological vector of *R. felis*, molecular evidence identified this pathogen in a range of arthropods, suggesting a broader host spectrum [62,63]. Human infections have been reported in several countries, including a case in Italy, which was likely imported [64].

Additionally, *R. barbariae*, an emerging member of the spotted fever group (SFG) *Rickettsia*, was identified in *Rh. bursa*. Initially detected in *R. bursa* ticks in Portugal in 2006 and provisionally named *Rickettsia sp. PoTiRb169* [65], this pathogen has since been confirmed in ticks from several countries from Europe, Asia, and Africa. It was also identified in various tick species, including *Rh. annulatus* and *Rh. simus* [66,67], and genera such as *Amblyomma* and *Hyalomma* [33,68,69,70]. Beyond ticks, *R. barbariae* was amplified from the flea *Vermipsylla alakurt* [48,68]. These findings highlight the expanding range of arthropod hosts and potential vectors for this pathogen.

*Coxiella burnetii* was also detected in an *H. lusitanicum* tick. Although the transovarial transmission of *C. burnetii* in this tick species was documented, the role of ticks in the transmission of Q fever remains debated [71]. Domestic ruminants are considered the main source of human infections and the primary mode of transmission to humans is through the inhalation of aerosols contaminated with *C. burnetii* from infected animals [72,73].

Our findings, while based on a single positive case, reinforce the association between *H. lusitanicum* and *T. annulata*. Although *T. annulata* is not a zoonotic agent, its detection is noteworthy as it is the causative agent of tropical theileriosis, a disease with significant veterinary importance. This pathogen is widely distributed across southern Europe, North Africa, and Asia, placing an estimated 250 million cattle at risk of infection [74]. In Sicily, *T. annulata* exhibits a high prevalence among cattle, making bovine theileriosis one of the most common tick-borne diseases in the region, with outbreaks reported annually. A serological study conducted in Sicily between 2018 and 2019 documented seroprevalence rates in cattle farms ranging from 22% to 71% [75]. *Hyalomma lusitanicum* is one of the tick species implicated in the transmission of *T. annulata* [76], and it has been identified as the primary vector responsible for transmitting this pathogen to cattle in Sicily.

## 5. Conclusions

The study identified both established and potential vector species capable of transmitting pathogens to animals and humans, highlighting a diverse tick community. The detected species included *H. lusitanicum*, *Rh. sanguineus* s.l., *Rh. turanicus*, *Rh. bursa*, *Rh. Pusillus,* and *D. marginatus*. A significant infection rate with various *Rickettsia* species was observed among both questing and feeding ticks. Additionally, *C. burnetii* and *T. annulata* were identified in questing ticks.

Phylogenetic analysis revealed *R. slovaca* as the most frequently detected species. Other *Rickettsia* species identified included *R. felis*, *R. barbariae*, *R. massiliae*, *R. conorii*, and *Candidatus* R. shennongii. A key challenge in the study was distinguishing between *R. massiliae* and *Candidatus* R. shennongii. To confirm the presence of *Candidatus* R. shennongii and other potentially novel *Rickettsia* variants, future research should utilize larger sample sizes and additional genetic targets. The detection of potentially new species, such as *Candidatus* R. shennongii, underscores the importance of continuous surveillance and comprehensive molecular characterization of *Rickettsia* in tick populations.

The diversity of ticks and associated pathogens identified in the study area, combined with the presence of wildlife and novel host–pathogen relationships, has significant implications for the ecology of tick-borne diseases. These findings highlight a potential risk to both human and animal health, emphasizing the critical need for ongoing monitoring and targeted mitigation strategies.

## Figures and Tables

**Table 1:** PCR performed for the amplification of DNA from *Rickettsia* spp., *Anaplasma* spp., and Piroplasmids, and the real-time PCRs performed for the amplification of *Coxiella burnetii*, *Borrelia* spp., and Piroplasmids.

Pathogen	Gene Target	PCR Assay	Primer/Probe Sequences	Reference
*Rickettsia* spp.	*ompA*	Nested PCR	Rr190.70p 5′-ATGGCGAATATTTCTCCAAAA-3′- Rr190.701n 5′-GTTCCGTTAATGGCAGCATCT--3′ Rr190.602n 5′-AGTGCAGCATTCGCTCCCCCT-3′	[16]
*Rickettsia* spp.	*ompB*	Nested PCR	rompB OF 5′-GTAACCGGAAGTAATCGTTTCGTAA-3′ rompB OR 5′-GCTTTATAACCAGCTAAACCACC-3′ rompB SFG IF 5′-GTTTAATACGTGCTGCTAACCAA-3′ rompB SFG IR 5′-GGTTTGGCCCATATACCATAAG-3′	[17]
*Rickettsia* spp.	*gltA*	PCR	409D 5′-CCTATGGCTATTATGCTTGC-3′ 1258N ATTCCAAAAAGTACAGTGAACA-3′	[18]
*Anaplasma* spp.	*16S-rRNA*	Nested PCR	EE1 5′-TCCTGGCTCAGAACGAACGCTGGCGGC-3′ EE2 5′-AGTCACTGACCCAACCTTAAATGGCTG-3′ EE3 5′-GTCGAACGGATTATTCTTTATAGCTTGC-3′ EE4 5′-CCCTTCCGTTAAGAAGGATCTAATCTCC-3′	[19]
*Coxiella burnetii*	*IS1111*	Real-time PCR	sIS1pri F 5′- CGGGTTAAGCGTGCTCAGTAT-3′ sIS1pri R 5′- TCCACACGCTTCCATCACCAC- 3′ Tqpro sIS1 (5′-FAM/3′-BHQ1) 5′-AGCCCACCTTAAGACTGGCTACGGTGGAT-3′	[20]
*Coxiella burnetii*	*htpB*	PCR	Q3 5′-GGCAATCACCAATAAGGGCCG-3′ Q5 5′-GCGGGTGATGGTACCACAACA-3′	[21]
*Borrelia* spp.	*ospA*	Real-time PCR	Bor_OspA_F 5′- AATATTTATTGGGAATAGGTCTAA-3′ Bor_OspA_R 5′-CACCAGGCAAATCTACTGA-3′ Bor_OspA_TM (5′-FAM/3′-BHQ1) 5′-TTAATAGCATGYAAGCAAAATGTTAGCA-3′	[22]
Piroplasmids	*18S rRNA*	Real-time PCR	Bab18S_F 5′- CATGAACGAGGAATGCCTAGTATG- 3′ Bab18S_R 5′- CCGAATAATTCACCGGATCACTC–3′ Bab18S_Pr (5′-FAM/3′-BQ1) 5′- CCGAATAATTCACCGGATCACTC—3′	[23]
Piroplasmids	*18S rRNA*	PCR	PIROA 5′-AATACCCAATCCTGACACAGGG-3′ PIROB 5′-TTAAATACGAATGCCCCCAAC-3′	[24]

**Table 2:** Total ticks screened and positives identified.

Free-Living Tick Species	No. of Male Tick Count	No. of Female Tick Count	Positive/Total Number (Positive Percentage)	Tick-Borne Pathogens
*Hyalomma lusitanicum*	25	44	18/69 (26.1)	*Rickettsia massiliae* (2)
				*Rickettsia massiliae—Candidatus* Rickettsia shennongii (2)
				*Candidatus* Rickettsia shennongii (1)
				*Rickettsia slovaca* (10)
				*Rickettsia felis* (1)
				*Coxiella burnetii* (1)
				*Theileria annulata* (1)
*Rhipicephalus pusillus*	14	13	2/27 (7.4)	*Rickettsia massiliae* (1) *Candidatus* Rickettsia shennongii (1)
*Rhipicephalus bursa*	4	8	3/12 (25)	*Rickettsia barbariae* (3)
*Rhipicephalus sanguineus s.l.*	0	17	1/17 (5.9)	*Rickettsia conorii* (1)
*Rhipicephalus turanicus*	5	4	3/9 (33.3)	*Rickettsia massiliae* (1)
				*Rickettsia massiliae—Candidatus* Rickettsia shennongii (1)
				*Candidatus* Rickettsia shennongii (1)
*Dermacentor marginatus*	0	3	1/3 (33.3)	*Rickettsia slovaca* (1)
**Feeding Tick Species**			**Positive/Total Number (Positive Percentage)**	**Tick-Borne Pathogens**
*Hyalomma lusitanicum*	51	24	1/75 (1.3)	*Rickettsia massiliae* (1)
*Dermacentor marginatus*	0	2	1/2 (50)	*Rickettsia massiliae* (1)

**Table 3:** Tick species collected in the study and detected pathogens per collection site.

Collection Site	Tick Species	Positive/Total Number per Site (Positive Percentage; CI 95%)	Tick-Borne Pathogens
Site 1—Sede Landolina	*Hyalomma lusitanicum*	5/37 (13.5)	*Rickettsia massiliae* (1)
			*Rickettsia massiliae—Candidatus* Rickettsia shennongii *(2)*
			*Candidatus* Rickettsia shennongii (1)
			*Coxiella burnetii* (1)
	*Rhipicephalus pusillus*	0/7	
	*Rhipicephalus bursa*	0/6	
Site 2—Boschetto Airoldi	*Hyalomma lusitanicum*	1/7 (14.2)	*Rickettsia massiliae* (1)
	*Rhipicephalus pusillus*	2/17 (11.7)	*Rickettsia massiliae* (1)
			*Candidatus* Rickettsia shennongii (1)
	*Rhipicephalus bursa*	3/6 (50)	*Rickettsia barbariae* (3)
	*Rhipicephalus sanguineus s.l.*	1/14 (7.1)	*Rickettsia conorii* (1)
	*Rhipicephalus turanicus*	3/9 (33.3)	*Rickettsia massiliae* (1)
			*Rickettsia massiliae—Candidatus* Rickettsia shennongii (1)
			*Candidatus* Rickettsia shennongii (1)
Site 3—Gorgo Santa Rosalia	*Hyalomma lusitanicum*	12/25 (48)	*Rickettsia slovaca* (10)
			*Rickettsia felis* (1)
			*Theileria annulata (1)*
	*Rhipicephalus pusillus*	0/3	
	*Rhipicephalus sanguineus s.l*	0/3	
	*Dermacentor marginatus*	1/3 (33.3)	*Rickettsia slovaca* (1)
Wild boar carcasses	*Hyalomma lusitanicum*	1/75 (1.3)	*Rickettsia massiliae* (1)
	*Dermacentor marginatus*	1/2 (50)	*Rickettsia massiliae* (1)

**Table 4:** Comparison of obtained sequences to the NCBI nucleotide database by BLAST.

Sample Id	Target	Taxon	Blast Id	Country of Origin	Reference	Blast Id %
MP-IV	*ompB*	*R. felis*	OM681612	India	[28]	99%
MP-III tick3 *	*ompB*	*R. slovaca*	MK301607	Spain	[29]	100%
MP-III tick4	*ompB*	*R. slovaca*	MK301607	Spain	[29]	100%
MP-III tick5	*ompB*	*R. slovaca*	MK301607	Spain	[29]	100%
MP-III tick6	*ompB*	*R. slovaca*	MK301607	Spain	[29]	100%
MP-III tick8	*ompB*	*R. slovaca*	MK301607	Spain	[29]	99%
MP-III tick10	*ompB*	*R. slovaca*	MK301607	Spain	[29]	100%
MP-III tick13	*ompB*	*R. slovaca*	MK301607	Spain	[29]	99%
MP-I tick17	*ompB*	*R. massiliae*	MN853118/CP000683	Portugal	[30,31]	99%
MP-I tick25	*ompB*	*R. massiliae—Candidatus* R. shennongii	ON646173/ON015827	Taiwan	[26,32]	100/99%
MP-I tick26 *	*ompB*	*R. massiliae—Candidatus* R. shennongii	ON646173/ON015827	Taiwan	[26,32]	100/99%
MP-I tick27	*ompB*	*R. massiliae—Candidatus* R. shennongii	ON646173/ON015827	Taiwan	[26,32]	99/99%
MP-I tick35	*ompB*	*R. massiliae*	MN853118/CP000683	Portugal	[30,31]	99%
MP-I tick36	*ompB*	*R. massiliae*	MN853118/CP000683	Portugal	[30,31]	99%
MP-I tick46	*ompB*	*R. massiliae*	MN853118/CP000683	Portugal	[30,31]	99%
MP-I tick61	*ompB*	*Candidatus* R. barbariae	KY233287	Lebanon	[33]	100%
MP-II tick17-1	*ompB*	*R. massiliae*	MN853118/CP000683	Portugal	[30]	99%
MP-II tick16-2	*ompB*	*R. massiliae*	MN853118/CP000683	Portugal	[30]	99%
MP-III tick2	*ompA*	*R. slovaca*	OP729880	Spain	[29]	100%
MP-III tick3 *	*ompA*	*R. slovaca*	OP729880	Spain	[29]	99%
MP-III tick7	*ompA*	*R. slovaca*	OP729880	Spain	[29]	99%
MP-III tick9	*ompA*	*R. slovaca*	OP729880	Spain	[29]	100%
MP-III tick14	*ompA*	*R. slovaca*	OP729880	Spain	[29]	99%
MP-I tick26 *	*ompA*	*Candidatus* R. shennongii	OL856103	China	[26]	99%
MP-I tick28	*ompA*	*Candidatus* R. shennongii	OL856103	China	[26]	99%
MP-I tick29	*ompA*	*Candidatus* R. shennongii	OL856103	China	[26]	99%
MP-I tick38	*ompA*	*R. conorii*	KY069258	China	[34]	98%
MP-I tick42	*ompA*	*Candidatus* R. shennongii	OL856103	China	[26]	100%
MP-I tick64	*ompA*	*Candidatus* R. barbariae	KY233249	Lebanon	[33]	100%
MP-I tick65	*ompA*	*Candidatus* R. barbariae	KY233249	Lebanon	[33]	100%

* Sample analyzed by both targets.

## Data Availability

Data are contained within the article.
